# Involvement of ClC-3 chloride/proton exchangers in controlling glutamatergic synaptic strength in cultured hippocampal neurons

**DOI:** 10.3389/fncel.2014.00143

**Published:** 2014-05-23

**Authors:** Raul E. Guzman, Alexi K. Alekov, Mikhail Filippov, Jan Hegermann, Christoph Fahlke

**Affiliations:** ^1^Institute of Complex Systems, Zelluläre Biophysik (Institute of Complex Systems-4), Forschungszentrum JülichJülich, Germany; ^2^Institut für Neurophysiologie, Medizinische Hochschule HannoverHannover, Germany; ^3^Laboratory for Brain Extracellular Matrix Research, University of Nizhny NovgorodNizhny Novgorod, Russia; ^4^Institut für Funktionelle und Angewandte Anatomie, Medizinische Hochschule HannoverHannover, Germany

**Keywords:** ClC-3, mEPSC, EPSC, γ-DGG, neurons

## Abstract

ClC-3 is a member of the CLC family of anion channels and transporters that localizes to early and late endosomes as well as to synaptic vesicles (SV). Its genetic disruption in mouse models results in pronounced hippocampal and retinal neurodegeneration, suggesting that ClC-3 might be important for normal excitatory and/or inhibitory neurotransmission in central neurons. To characterize the role of ClC-3 in glutamate accumulation in SV we compared glutamatergic synaptic transmission in cultured hippocampal neurons from WT and *Clcn3-/-* mice. In *Clcn3-/-* neurons the amplitude and frequency of miniature as well as the amplitudes of action-potential evoked EPSCs were significantly increased as compared to WT neurons. The low-affinity competitive AMPA receptor antagonist γ-DGG reduced the quantal size of synaptic events more effectively in WT than in *Clcn3-/-* neurons, whereas no difference was observed for the high-affinity competitive non-NMDA antagonist NBQX. Paired pulse ratios of evoked EPSCs were significantly reduced, whereas the size of the readily releasable pool was not affected by the genetic ablation of ClC-3. Electron microscopy revealed increased volumes of SV in hippocampi of* Clcn3-/-* mice. Our findings demonstrate that ClC-3 controls fast excitatory synaptic transmission by regulating the amount of neurotransmitter as well as the release probability of SV. These results provide novel insights into the role of ClC-3 in synaptic transmission and identify excessive glutamate release as a likely basis of neurodegeneration in *Clcn3-/-*.

## INTRODUCTION

Fast synaptic transmission in the mammalian central nervous system is initiated by the exocytosis of neurotransmitters from the presynaptic nerve terminal. The specificity and efficiency of synaptic transmission relies on selective and effective accumulation of neurotransmitters into synaptic vesicles (SV) by vesicular secondary-active transporters. Vesicular neurotransmitter transporters utilize the electrochemical gradient for protons (Masson et al., 1999) that is generated by vacuolar ATPases (V-ATPases; Finbow and Harrison, 1997; Takamori, 2006). V-type ATPases are electrogenic, and effective acidification of SV thus requires the additional transport of counter ions to prevent excessive depolarization and to decrease energy demand of proton pumping. Isolated vesicles usually only acidify in the presence of small anions such as Cl^-^ (Xie et al., 1983), and anion channels or transporters are generally assumed to support V-ATPase by maintaining electroneutrality in intracellular organelles.

ClC-3 is a member of the CLC family of anion channels and transporters (Jentsch, 2008) that is expressed in various regions of the central nervous system. Its localization to SV (Stobrawa et al., 2001; Gronborg et al., 2010) makes ClC-3 a candidate anion transporter necessary for effective neurotransmitter accumulation. Genetic disruption of ClC-3 results in severe central neurodegeneration (Stobrawa et al., 2001; Dickerson et al., 2002; Yoshikawa et al., 2002). This phenotype suggests excessive glutamate release in the absence of ClC-3, however, a recent study did not observe significant differences in excitatory synaptic transmission between WT and *Clcn3-/-* mice (Stobrawa et al., 2001), most likely due to neurodegeneration and secondary downregulation of vesicular glutamate transporters in the studied *Clcn3-/-* animals.

To prevent potential interferences of neurodegeneration in studying glutamatergic synaptic transmission in *Clcn3-/-* mice we used cultured hippocampal neurons for our experiments. The comparison of miniature and evoked excitatory postsynaptic currents (EPSC) as well as the ultrastructural analysis of SV in WT and *Clcn3-/-* neurons demonstrates that ClC-3 modulates the magnitude of synaptic events by altering the size and glutamate content as well as the release probability of SV.

## MATERIALS AND METHODS

### CELL CULTURE

We prepared dissociated cultures from hippocampal pyramidal neurons from WT or *Clcn3-/-* mice (kindly provided by Dr. Thomas Jentsch) at postnatal day 1 as described previously (Guzman et al., 2010). Since *Clcn3-/-* mice show selective degeneration of the hippocampus starting at an age of about 3 weeks (Stobrawa et al., 2001), we performed our experiments on culture days 14–15 to avoid alterations of neuronal function by potential ultrastructural alterations.

### ELECTROPHYSIOLOGY

Whole-cell voltage clamp recordings were performed on pyramidal neurons from WT or *Clcn3-/-* neurons (Guzman et al., 2010) using an Axopatch 200B amplifier (Molecular Devices, Sunnyvale, CA, USA). Patch pipettes with resistances between 3 and 4 MΩ were filled with intracellular solution containing (in millimolars) 137.5 K-gluconate, 11 NaCl, 2 MgATP, 0.2 Na_2_GTP, 1.1 EGTA, 11 HEPES, 11 D-glucose, pH 7.3. Only cells with access resistances of 6–10 MΩ were analyzed, and 80–85% of the access resistance was compensated. Currents were filtered at 5 kHz and digitized at 50 kHz. The standard extracellular solution consisted of (in millimolars) 130 NaCl, 10 NaHCO_3_, 2.4 KCl, 4 CaCl, 4 MgCl_2_, 10 HEPES, 10 D-glucose, pH 7.4 with NaOH. The osmolarity of intra- and extracellular solution was adjusted to 310 mOsm with D-glucose. Action potential-evoked release was studied after addition of 25 μM bicucullin to the extracellular solution. For experiments characterizing miniature EPSCs (mEPSCs) the extracellular solution was supplemented with 1 μM tetrodotoxin (Sigma-Aldrich, Schnelldorf, Germany), 25 μM bicucullin (Tocris Bioscience, Ellisville, MI, USA) and 25 μM APV [(2*R*)-amino-5-phosphonovaleric acid; (2*R*)-amino-5-phosphonopentanoate; Tocris Bioscience, Ellisville, MI, USA].

Quantal signals were recorded by clamping neurons to –70 mV for periods of 60 s. mEPSCs were detected as spontaneous events with peak amplitudes >15 pA (~5 times the S.D. of the background noise, e.g., WT: 3.2 ± 0.08 pA, *Clcn3-/-*; 3.05 ± 0.09 pA, *n* = 5) and total charges – estimated as integral over the mEPSC >25 fC were analyzed using a commercial software (Mini analysis, Synaptosoft, Version 6.0.3, Decatur, GA, USA). Action potential-evoked EPSCs were recorded using an EPC 10 amplifier controlled by PatchMaster (HEKA, Lambrecht, Pfalz, Germany) software. Cells were clamped to -70 mV, and EPSCs were elicited from hippocampal neurons by local extracellular stimulation as described (Maximov et al., 2007a). Synaptic responses were triggered by 0.5 mA/1 ms current injection at frequency of 0.2 Hz via bipolar electrode (PI2CEA3 concentric bipolar electrode, tip diameter 2–3 μm platinum/iridium, Hofheim, Germany ) placed at a distance of 200–250 μM from the patched cell. For paired-pulse experiments, neurons were stimulated by pairs of extracellular current application with 20 ms interstimulus interval. Peak current amplitudes were measured from baseline current amplitudes determined before the stimulation. EPSCs – evoked by 1 ms stimuli and- usually had a time-to-peak duration of about 2 ms. Artifacts and current peak amplitudes are thus normally easy to separate (Guzman et al., 2010). Moreover, possible contaminations of synaptic currents by stimulation artifacts can be easily detected by the different time courses of EPSC and artifact. We carefully checked all experiments for deviations of measured EPSCs from the typical time course with rapid rise time (20–80% rise time of 0.26–0.50 ms) and decay time constants of 4.6–10.0 ms. EPSCs were analyzed using FitMaster (HEKA) and Origin (OriginLab, USA) software. For comparison of action potential-evoked postsynaptic currents in WT and *Clcn3-/-* cultures were alternatingly studied at the same setup with the bipolar electrode set to the same distance from the cell body for all evaluated cells. To estimate the size of the readily releasable pool (RRP) we measured synaptic responses evoked by the fast application of hypertonic solution for 5s (500 mM sucrose) using a gravity-fed fast flow system. To test for possible differences in postsynaptic glutamate receptor sensitivity we evoked postsynaptic currents in WT and *Clcn3-/-* cultures by applying 100 μM L-glutamate glutamate using a FemtoJet perfusion system (Eppendorf, Germany). In these experiments a custom-made perfusion pipette was positioned close to the recording patch-pipette and glutamate was applied for 100 ms. Experiments were performed in the absence as well as in the presence of 200 μM γ-DGG added to the bath solution.

All experiments were performed with at least three different preparations with WT and *Clcn3-/-* mice, and all comparisons were between cultures from WT and *Clcn3-/-* littermates. All experiments were performed at room temperature (22–23°C).

### ELECTRON MICROSCOPY

After transferal to a 200 μm deep aluminum platelet (Microscopy Services, Flintbek, Germany) fresh hippocampal tissues were frozen using a Leica HPM 100 (Wetzlar, Germany), followed by freeze substitution in 0.1% tannic acid at -90°C for 48 h in a Leica AFS2 (Wetzlar, Germany). Solutions were then changed to 2% OsO4, and the temperature was raised initially to -20°C and after 7 h to 4°C. Samples were washed in acetone and embedded in EPON. Fifty nanometers ultrathin sections were post-stained with 4% uranyl acetate and lead citrate (Reynolds, 1963), mounted onto form var coated copper grids, stained with 4% uranyl acetate and lead citrate (Reynolds, 1963) and then examined in a Morgagni TEM (FEI, Hillsboro, OR, USA), operated at 80 kV. Images from different tissues blocks of the same animal were taken with a 2K side mounted Veleta CCD camera, binned to 1024 × 1024 pixels. Experiments were performed on hippocampi of at least three different WT and *Clcn3-/-* animals.

### IMMUNOFLUORESCENCE

Neuronal cultures were blocked and permeabilized with 5% goat serum (Sigma-Aldrich, Schnelldorf, Germany ) and 1% Triton X100 for 45 min after fixation in PBS containing 4% paraformaldehyde at room temperature. Primary antibodies against MAP2 (rabbit polyclonal, Synaptic Systems, Göttingen, Germany) and anti-VGLUT1 (mouse monoclonal, Synaptic Systems, Göttingen, Germany) were diluted in 1% goat serum and 0.1% Triton X100 in PBS and then added to cells for 60 min. Subsequently, a secondary antibody linked to Alexa-Fluor 633 and 488 (Invitrogen, Darmstadt, Germany) was applied for 60 min. After washing in PBS, coverslips containing cells were either imaged immediately or mounted on a glass slide with 2 μl Fluoromount-G (SouthernBiotech).

Images were acquired using a Leica DM IRD (Wetzlar, Germany) inverted microscope equipped with a 63× oil objective and analyzed with NIH imageJ 1.45. Immuno-positive spots were determined using a threshold-based detection routine with a threshold adjusted to dendritic background signals. Immunosignals were quantified as mean fluorescent intensity, and synaptic density was determined by counting the number of VGLUT1-stained puncta per 50 μm dendrite length identified by MAP2 staining.

### QUANTIFICATION OF PROTEIN EXPRESSION LEVELS

Cell surface expression of GluR1 receptors was assayed with a modification of cell surface biotinylation methods (Du et al., 2004). Hippocampal cultures obtained from *Clcn3-/-* mice and WT litter mate were washed with ice-cold PBS (with calcium and magnesium pH 7.4; Invitrogen, Darmstadt, Germany) to prevent receptor internalization. After three washes with PBS cells were incubated with sulfo-NHS-LC biotin (0.25 mg/ml in ice-cold PBS) (ThermoScientific, Rockford, IL, USA) for 30 min. The reaction was stopped by removal of the above solution and incubation for 20 min in ice-cold PBS containing 10 mM glycine. After threefold washing and lysis with RIPA buffer [in millimolars): 20 HEPES, 100 NaCl, 1 EGTA, 1 Na-orthovanadate, 50 NaF, protease inhibitor (Roche), 1% NP40, 1% deoxicholate, 0,1% SDS pH 7.4], biotinylated proteins were precipitated with immune pure immobilized streptavidin (Pierce, ThermoScientific, Rockford, IL, USA). Biotinylated proteins were separated in 10% SDS-PAGE gel and transferred to nitrocellulose membrane. Membranes were probed with an anti-GluR1 antibody (rabbit polyclonal, 1:700, abcam, Cambridge, UK) or anti-actin (rabbit, poloclonal, 1:1000, sigma, St. Louis, MO, USA) followed by peroxidase-conjugated goat anti-rabbit IgG (1:25000, Invitrogen, Darmstadt, Germany). Immunoreactive bands were visualized by enhanced chemiluminiscence (ECL, AppliChem, Darmstadt, Germany) using a GeneGnome chemiluminiscence imagine system (SYNGENE, Cambridge, UK) and ImageJ software.

We compared expression of VGLUT1 mRNA levels in cultured neurons from four different WT and *Clcn3-/-* preparations. Total RNA was prepared using the PureLink RNA Mini Kit (Ambion, Darmstadt, Germany ). Reverse transcription was performed from 0.1 μg RNA using the High Capacity cDNA Reverse Transcription Kit (Applied Biosystems, Darmstadt, Germany), and VGLUT1 mRNA was quantified with the 2^-Δ^^Δ^^CT^ method using a specific TaqMan Assay for VGLUT1 (Mm00812886_m1, StepOnePlus System, Applied Biosystems, Darmstadt, Germany).

### STATISTICAL ANALYSIS

All summary data are given as mean ± SEM. Paired Student’s *t* analysis was used to test statistical differences with ^*^p <0.05, ^**^p <0.01, ^***^p < 0.001 levels of significance. Authors warrant that any human and/or animal studies have been approved by an appropriate institutional review committee.

## RESULTS

### INCREASED MINIATURE EPSC AMPLITUDES IN *Clcn*3**-/-** NEURONS ARE CAUSED BY ALTERED SYNAPTIC GLUTAMATE CONCENTRATIONS

**FIGURE 1:**
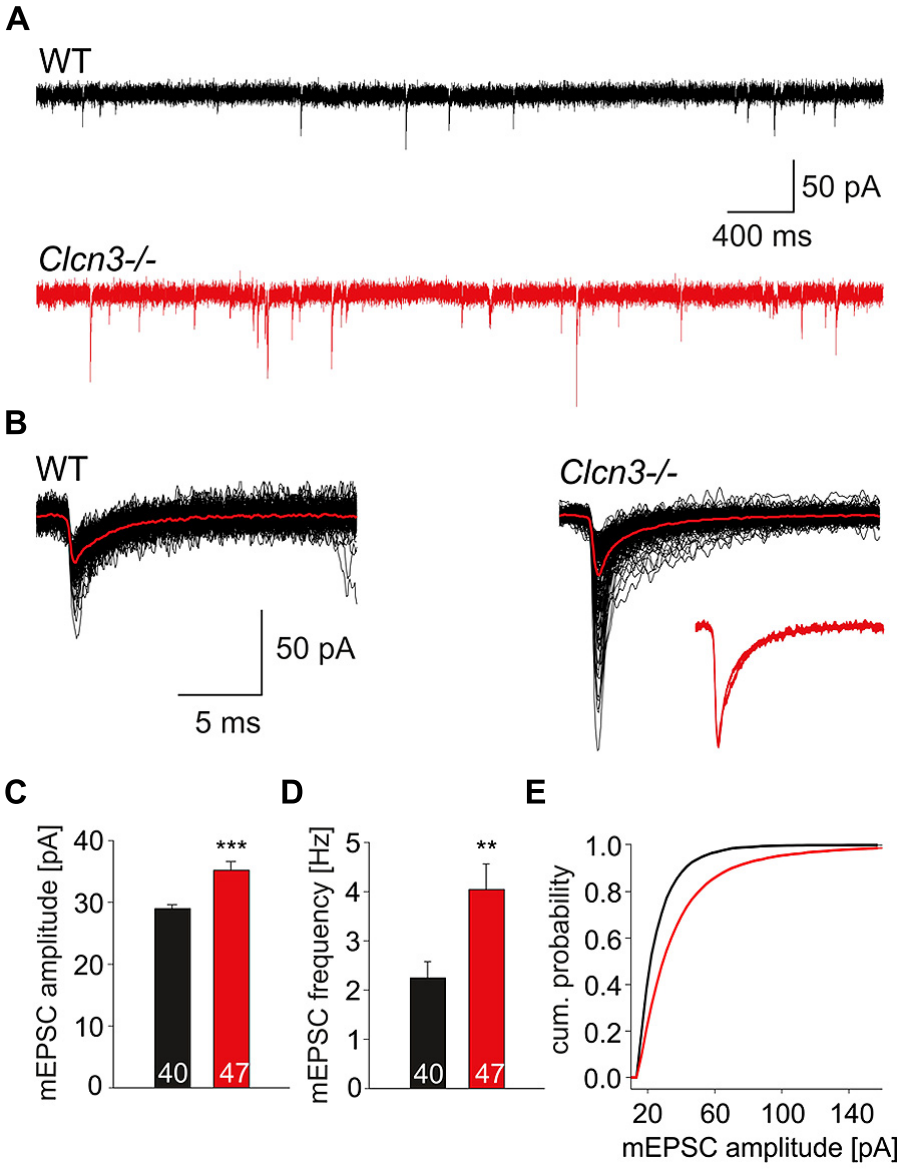
**Quantal glutamatergic signals are altered in *Clcn3-/-* neurons. (A)** Representative mEPSCs from WT and *Clcn3-/-* neurons at a holding potential of -70 mV. **(B)** Superimposed representative spontaneous events recorded from WT or *Clcn3-/-* neurons. Averaged quantal events are shown in red, and a comparison of averaged normalized mEPSCs from WT and *Clcn3-/-* neurons are shown as inset. **(C)** mEPSC peak amplitudes and **(D)** frequencies from *Clcn3-/-* and WT neurons. Data are given as mean ± SEM from at least five different cultures. **(E)** Cumulative amplitude frequency distribution for WT (black line) and *Clcn3-/-* (red line) neurons. (***p < 0.01 and **p < 0.001 *t*-student), numbers in the bars represent the number of evaluated cells.

**Figure 1** shows representative whole-cell patch clamp recordings from miniature excitatory postsynaptic currents from WT and *Clcn3-/-* neurons. Cells were held at -70 mV, and quantal signals were acquired for a period of 60 s in the presence of 1 μM TTX, 25 μM APV and 25 μM bicuculline. Miniature excitatory postsynaptic currents (**Figure 1A**) display comparable time courses in WT as well as in *Clcn3-/-* neurons (**Figure 1B**, insert), but distinct amplitudes (**Figure 1C**; WT 28.9 ± 0.6 pA, *n* = 40 and* Clcn3-/-* 35.2 ± 1.4 pA, *n* = 47, *p* < 0.001) and frequencies (**Figure 1D**; WT 2.2 ± 0.3 Hz, *n* = 40 and* Clcn3-/-* 4.0 ± 0.5 Hz, *n* = 47, *p* < 0.01) as illustrated by the amplitude frequency distribution in **Figure 1E**.

**FIGURE 2:**
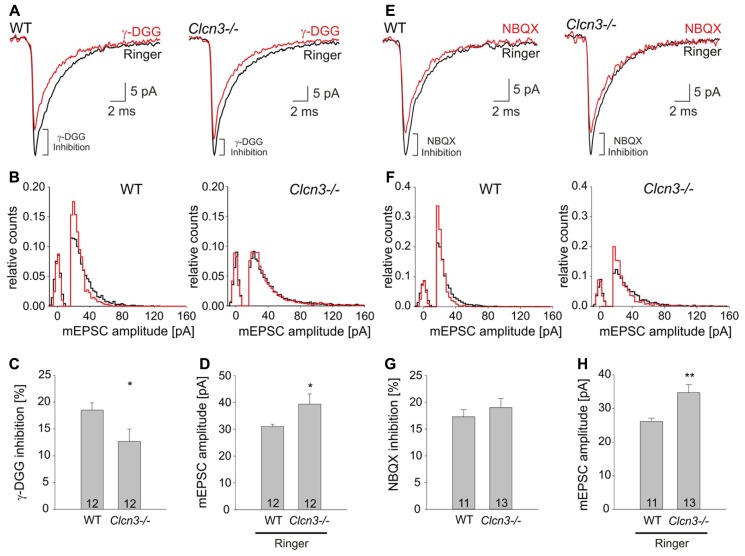
**The low-affinity competitive AMPA receptor antagonist γ-DGG blocks mEPSC amplitudes more efficiently in WT than in *Clcn3-/-* neurons. (A)** Ensemble mEPSC averages from WT (left) or *Clcn3-/-* (right) neurons recorded in the absence (black trace) or in the presence of γ-DGG (red trace). **(B)** Peak mEPSC amplitude distributions for WT (left) and *Clcn3-/-* (right), in the absence (black) or in the presence of γ-DGG (red). **(C)** Relative γ-DGG induced mEPSC amplitude reductions for WT and *Clcn3-/-*. **(D)** mEPSC amplitudes from WT and *Clcn3-/-* neurons in the absence of γ-DGG. **(E)** Ensemble mEPSCs average from WT (left) and *Clcn3-/-* (right) cells recorded in the absence (black trace) or in the presence of NBQX (red trace). **(F)** Peak amplitude distributions of WT (left) and *Clcn3-/-* (right) mEPSCs in the absence (black) or in the presence of NBQX (red). **(G)** WT and *Clcn3-/-* mEPSC peak amplitudes are similarly sensitive to NBQX in **(H)** mEPSC amplitudes from WT and *Clcn3-/-* neurons in the absence of NBQX. Data are given as means ± SEM from at least three different cultures (**p < 0.01 and *p < 0.05 *t*-student) numbers in the bar represent the number of evaluated cells.

The observed effects on quantal signals might be caused by changes in the glutamate concentration in the synaptic cleft after exocytosis of individual glutamatergic vesicles. To probe ClC-3-dependent alterations in the synaptic glutamate concentration we took advantage of γ-DGG, a rapidly dissociating competitive antagonist of AMPA receptors which block AMPA receptors with lower efficacy at higher glutamate concentrations (Liu et al., 1999). We found that application of 200 μM γ-DGG reduces the mean peak mEPSC amplitude at WT and mutant neurons, however, the effect is less pronounced in the *Clcn3-/-* than in WT (**Figure 2A**). The cumulative frequency distribution of mEPSC amplitudes revealed that γ-DGG shifted the distribution of events towards lower values in WT than in *Clcn3-/-* neurons (**Figure 2B**). The efficiency of the blocker in reducing the mean peak mEPSC amplitude was ~1.5 fold stronger for WT than for *Clcn3-/-* neurons, as expected for higher glutamate concentration in *Clcn3-/-* than in WT synaptic clefts. We found that application of γ-DGG reduces mean peak mEPSC amplitudes by 18 ± 2 % for WT (*n* = 12, three different cultures), but only by 12 ± 2% (*n* = 12, three different cultures) for *Clcn3-/-* neurons (*p* < 0.05; **Figure 2C**).

1,2,3,4-Tetrahydro-6-nitro-2,3-dioxo-benzo[f] quinxaline-7-sulfonamide (NBQX) is known to block non-NMDA receptors to the same extent regardless of the glutamate concentration (Liu et al., 1999). Application of NBQX to WT or* Clcn3-/-* cultures attenuated quantal signals (**Figure 2E**), and shifted the mEPSC amplitude cumulative frequency distribution to the left (**Figure 2F**). However, in contrast to γ-DGG, NBQX inhibition was not different between WT and* Clcn3-/-* (**Figure 2G**; mean peak mEPSC amplitudes reduction for WT 17.3 ± 1.4%, *n* = 11 and *Clcn3-/-* 19.0 ± 1.7,* n* = 13, *p* = 0.22, three different cultures). **Figures 2D,H** depict control mean mEPSC amplitudes from the cultures used in these experiments, illustrating consistently increased quantal signals in *Clcn3-/-* neurons in the absence of blockers. Taken together, our results indicate that synaptic cleft glutamate concentrations are higher in *Clcn3-/-* than in WT neurons.

## ALTERED EVOKED NEUROTRANSMITTER RELEASE IN *Clcn3-/-* NEURONS

**FIGURE 3:**
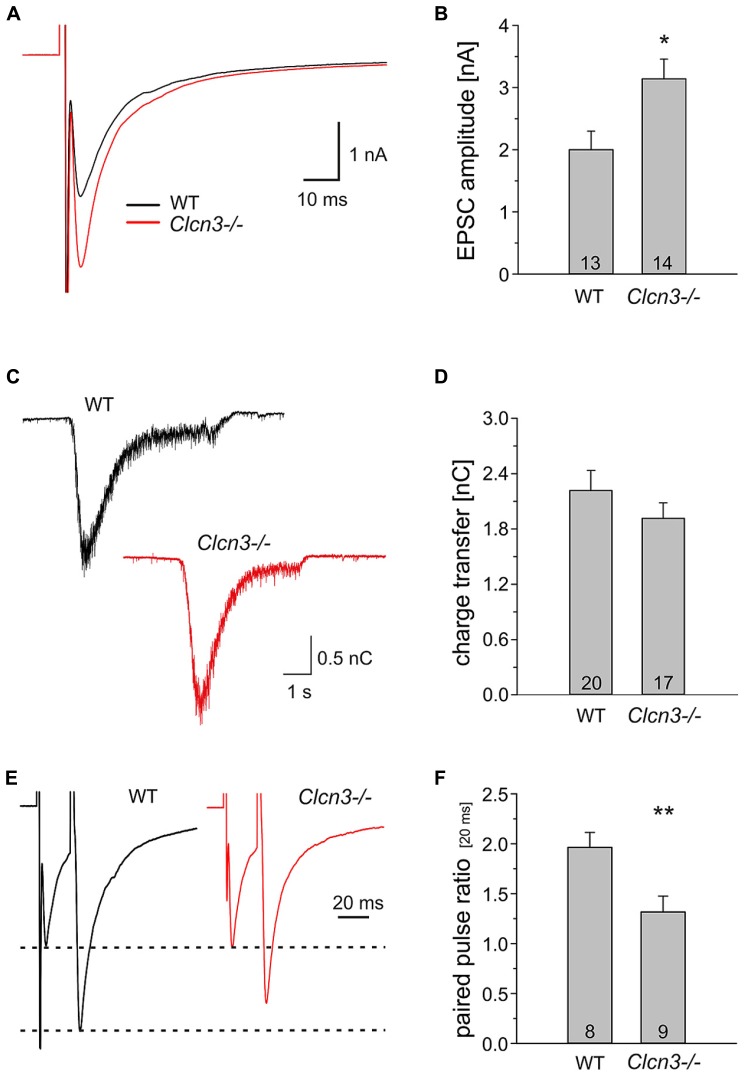
**Evoked synaptic signals are larger in *Clcn3-/-* than in WT neurons. (A)** Averaged evoked EPSCs from hippocampal neurons from WT (black trace) or *Clcn3-/-* neurons (red trace). **(B)** Mean EPSC amplitudes from WT and *Clcn3-/-* neurons. **(C) **Representative secretory responses to stimulation with 500 mOsm sucrose from WT (black trace) or *Clcn3-/-* neurons (red trace). **(D)** Mean ± SEM for readily releasable pool (RRP) charges for WT and *Clcn3-/-* neurons. The RRP charge is defined by the time integral over the first 1.2 s after the onset of the sucrose response. **(E)** Averaged paired-pulse EPSC traces from WT (black trace) and *Clcn3-/-* (red trace) at an inter-stimulus interval of 20 ms. **(F)** Mean ± SEM from paired-pulse ratios for WT or *Clcn3-/-* neurons. (**p < 0.01 and *p < 0.05 *t*-student), numbers in bars represent the number of analyzed cells.

To test whether the absence of ClC-3 also affects evoked synaptic responses we studied postsynaptic currents after local extracellular stimulation using a concentric bipolar electrode (Maximov et al., 2007a,b). Upon low frequency electrical stimulation (0.2 Hz) action potential-evoked responses have significantly higher amplitudes in *Clcn3-/-* than in WT neurons (WT 2.0 ± 0.3 nA, *n* = 13 and* Clcn3-/-* 3.1 ± 0.3 nA, *n* = 14, *p* = 0.03; **Figures 3A,B**).

Evoked EPSCs were even increased to a larger extent than quantal signals in *Clcn3-/-* neurons. This result is in agreement with the twofold higher mEPSC frequency and suggests that the size of the RRP of SV and/or released probability might be altered in the *Clcn3-/-* neurons. We first tested whether genetic ablation of ClC-3 affects the size of the RRP of SV. There are functionally distinct fractions of SV within presynaptic nerve terminals. One fraction, the so-called RRP of vesicles (Rizzoli and Betz, 2005), encompasses vesicles that are close to release sites and fuse and release their content first during nerve activity. Vesicles belonging to the RRP also fuse upon application of hypertonic solution (Rosenmund and Stevens, 1996). Hypertonic shock is the most frequently used and most established technique to quantify the RRP in cultured hippocampal neurons (Rizzoli and Betz, 2005). Moreover, recent work demonstrated that estimation of the RRP size either by tetanic stimulation or by hypertonic challenge provides similar results (Stevens and Williams, 2007). We determined the size of RRP as the integral of the synaptic current elicited by the application of 500 mM of sucrose for 5s (**Figure 3C**) and found no difference between WT and* Clcn3-/-* (WT 2.2 ± 0.2 nC, *n* = 20 and* Clcn3-/-* 1.9 ± 0.2 nC, *n* = 17, *p* = 0.15; **Figure 3D**).

To examine the release probability in WT and* Clcn3-/-* neurons we used paired-pulse stimulation, i.e., the application of two pulses in quick succession. In such experiments, high paired pulse ratio (PPR, the ratio of the amplitude of the second pulse to that of the first) indicate low probability of release, whereas synapses exhibiting lower PPRs are considered to have higher release probability. We detected a significant reduction in pair pulse ratio (20 μs interval) in *Clcn3-/-* neurons when compared to WT (WT 2 ± 0.16 PPR, *n* = 8 and* Clcn3-/-*1.3 ± 0.15 PPR, *n* = 9, *p* = 0.01; **Figures 3E,F**). We conclude that the absence of ClC-3 increases not only the glutamate content, but also the release probability of SV.

### CLC-3 REGULATES THE SIZE OF SYNAPTIC VESICLES

**FIGURE 4:**
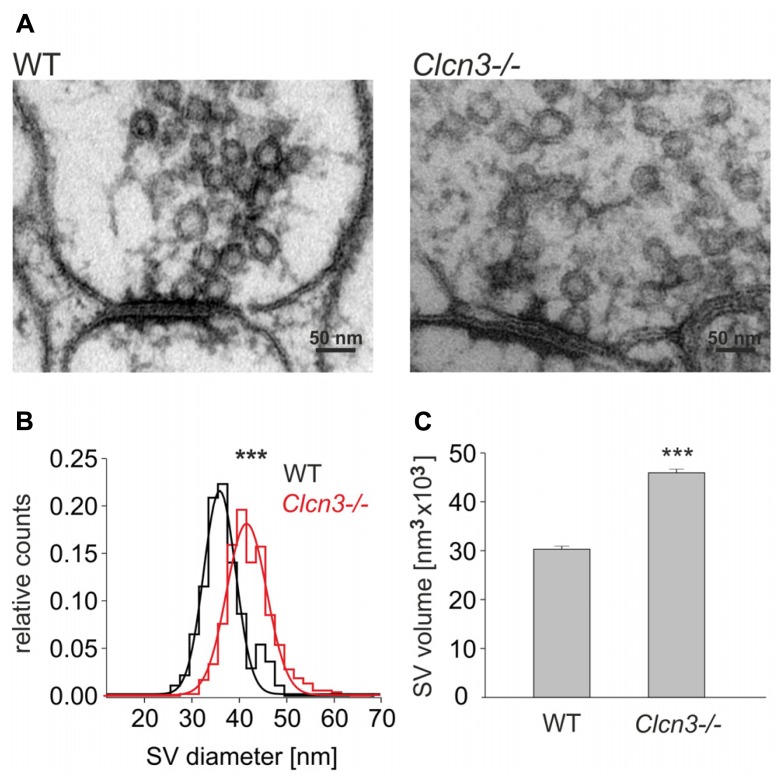
**ClC-3 regulates synaptic vesicle size. (A)** Representative electron micrographs for WT and *Clcn3-/-* synaptic terminals. **(B)** Distribution for synaptic vesicle diameters from WT (black) and *Clcn3-/-* (red) neurons. **(C)** Mean synaptic vesicle volumes from WT or *Clcn3-/-* neurons. Vesicle volumes were calculated from measured diameters assuming a spherical shape. Means ± SEM from 350 WT and 550 *Clcn3-/-* synaptic vesicles, three independent preparation (^***^p < 0.001, *t*-student).

Enhanced glutamate release might be associated with enlarged SV in *Clcn3-/-* neurons. We performed an ultrastructural analysis of the size of SV at synapses in hippocampal slices from WT and *Clcn3-/-* neurons (**Figures 4A,B**). Synapses can be readily identified by a heavily stained postsynaptic density in close proximity to presynaptic terminals filled with clusters of SV. Synaptic vesicle sizes were estimated by measuring the outer vesicle diameter in different blocks of the same animal from at least three independent preparation, with mean values of WT vesicles of 38.2 ± 0.2 nm (*n* = 350 SV, 22 synaptic terminals) and of 43.8 ± 0.2 nm in *Clcn3-/-* neurons (*n* = 550 SV, 15 synaptic terminals, *p* < 0.001). The distribution of synaptic vesicle outer diameters exhibits a significant shift towards larger values in *Clcn3-/-* neurons (**Figure 4B**). Assuming a spheroidal geometry of SV these values correspond to 1.5 fold increased vesicle volumes in knock-out as compared to WT neurons (**Figure 4C**). The increased magnitude of synaptic events in *Clcn3-/-* neurons thus corresponds morphologically to increased vesicle sizes.

### GENETIC ABLATION OF CLC-3 NEITHER AFFECTS THE NUMBER OF SYNAPSES NOR EXPRESSION OF AMPA RECEPTOR AND VESICULAR GLUTAMATE TRANSPORTERS

**FIGURE 5:**
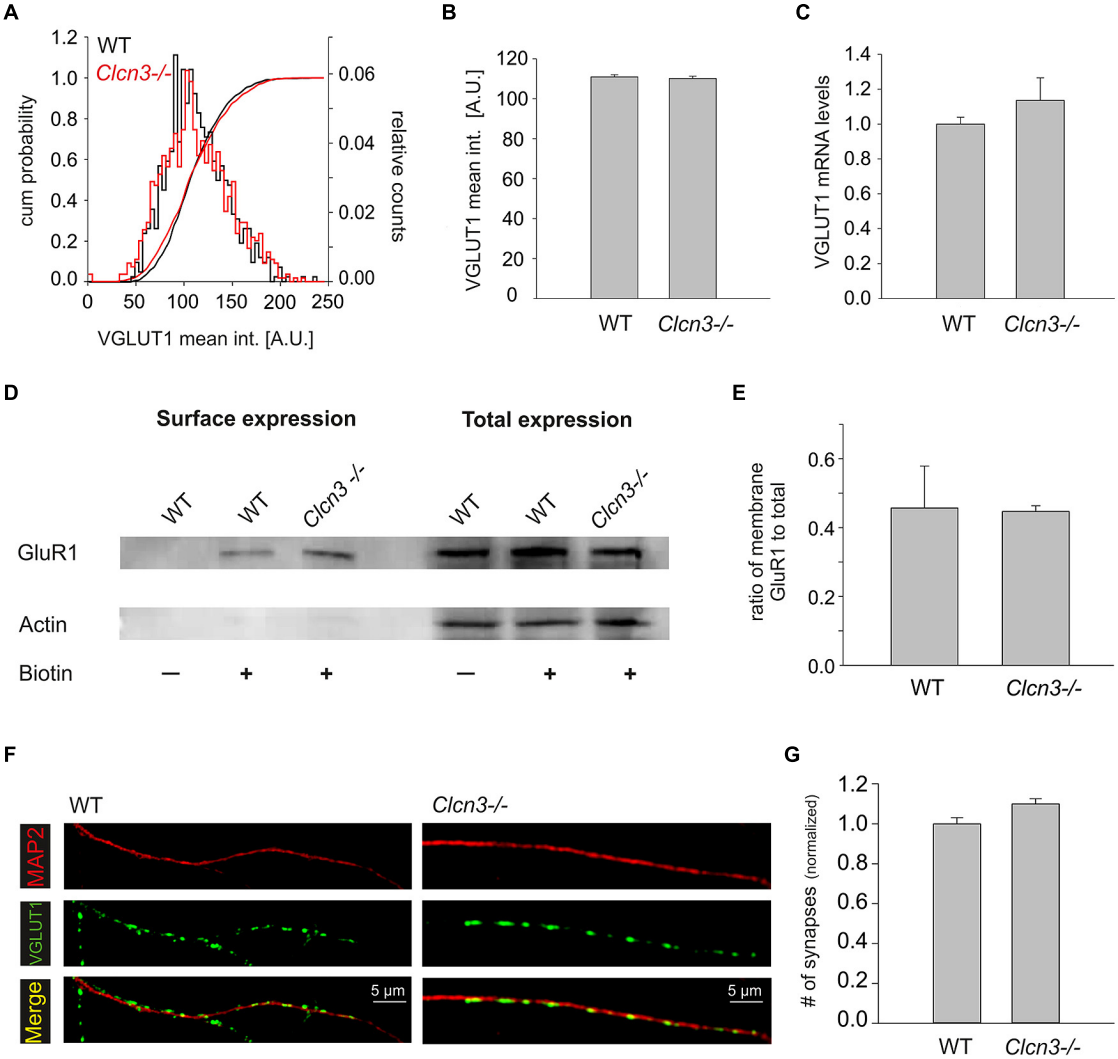
**Synaptogenesis and expression of AMPA receptors and VGLUT1 are not altered in *Clcn3-/-* neurons. (A)** Cumulative mean intensity distribution and superimposed cumulative intensity probability of VGLUT1 staining at synapses for WT and *Clcn3-/-.* **(B)** Mean fluorescence intensity for VGLUT1 staining from *Clcn3-/-* (*n* = 943 synapses) and WT (*n* = 724 synapses, *p* = 0.6, *t-*test) synapses. **(C)** Analysis of the VGLUT1 mRNA levels using quantitative qRT-PCR for WT and *Clcn3-/-* neurons (four different cultures, *p* = 0.52, *t*-student). **(D)** Surface and total expression levels of GluR1 in WT and *Clcn3-/-* neurons determined by biotinylation and western blot analysis. **(E)** Mean fraction of biotinlylated GluR1 from WT and *Clcn3-/-* neurons. Means ± SEM from at least four different cultures. **(F)** Representative epifluorescence images of WT and *Clcn3-/-* neurons after immunolabeling with antibodies to the vesicular glutamate transporter 1 (VGLUT1) and anti-MAP2. **(G)** Mean synapse density ± SEM quantified as number of VGLUT1 positive puncta per 50 μm dendrite length identified by MAP2 staining from WT and *Clcn3-/-* neuronal cultures (*n* = 17–20 per condition, *p* = 0.36, *t*-student).

Vesicular glutamate transporters (VGLUTs) are necessary for glutamate accumulation in SV, and the quantal size of excitatory postsynaptic currents is therefore critically dependent on expression levels of these transporters (Wilson et al., 2005). We stained WT and *Clcn3-/-* neurons with anti-VGLUT1 antibodies and estimated protein expression of VGLUT by determining intensity levels at distinct locations. No differences in event numbers or in cumulative frequency distributions of VGLUT1 intensities at synapses were found. (WT *n* = 724 synapses and* Clcn3-/- n* = 943 synapses, *p* = 0.6; **Figures 5A,B**). Moreover, mRNA levels of VGLUT1 were not significantly different between WT and *Clcn3-/-* neurons (four different cultures, *p* = 0.52; **Figure 5C**).

GluR1 is highly expressed in hippocampal pyramidal neurons and changes in its number are known to alter synaptic strength (Harms et al., 2005; Han and Stevens, 2009). We determined total expression levels and surface density of GluR1 AMPA receptor subunit using western blotting of full lysates and surface biotinylation without any discernible differences between WT and *Clcn3-/-* neurons (**Figures 5D,E**). We furthermore compared WT and *Clcn3-/-* postsynaptic currents evoked by direct application of 100 μM L-glutamate. No difference was observed between WT (2.9 ± 0.2 nA, *n* = 7) and *Clcn3-/-* (3.1 ± 0.6 nA, *n* = 6, *p* = 0.38) neurons. To exclude the possibility that saturation might have masked possible differences in receptor activation experiments were repeated in the presence of 200 μM of γ-DGG. As expected, evoked current in responses to 100 μM L-glutamate were smaller in the presence of 200 μM γ-DGG than the corresponding responses to 100 μM L-glutamate alone, but no difference was observed for WT (2.2 ± 0.2 nA, *n* = 6) and *Clcn3-/-* (1.9 ± 0.1 nA, *n* = 5, *p* = 0.2).

The frequency of mEPSCs critically depends on the number of synapses in our experimental system. We determined the numbers of synapses for WT and mutant neurons by identifying dendrites by MAP2 staining, and determining the density of synapses as number of VGLUT1-positive dots per 50 μm dendrites (**Figures 5F,G**). This approach provided similar synapse numbers for WT and *Clcn3-/-* (*p* = 0.36). Taken together, these results indicate that neither the number of synapses nor expression levels/sensitivity of AMPA receptors and VGLUTs differ in WT and *Clcn3-/-* neurons.

## DISCUSSION

We here used patch clamp recordings on cultured WT and *Clcn3-/-* neurons study the role of ClC-3 in glutamatergic synaptic transmission. We found that miniature (**Figure 1**) as well as evoked (**Figure 3**) excitatory postsynaptic currents exhibit significantly higher amplitude in *Clcn3-/-* as in WT neurons. Pharmacological approaches demonstrated higher glutamate concentration in *Clcn3-/-* than at WT synapses (**Figure 2**). These changes in glutamate release are not caused by altered VGLUT1 expression in our cultures (**Figures 5A–C**), and we thus conclude that ClC-3 regulates the amount of released glutamate by increasing the driving force for vesicular glutamate transport. We furthermore observed twofold increased mEPSC frequency (**Figure 1**), but unchanged synaptic density in *Clcn3-/-* cultures (**Figures 5F,G**). Paired pulse ratios were significantly reduced in *Clcn3-/-* as compared with the WT neurons (**Figures 3E,F**), whereas the size of the RRP was not affected (**Figures 3C,D**) by the genetic ablation of ClC-3. Taken together, these results indicate that ClC-3 increases the likelihood of synaptic vesicle fusion without interfering with the establishment of the vesicle’s release-ready state.

Earlier experiments revealed no significant differences in mean amplitudes of miniature EPSCs or IPSCs from *Clcn3-/-* and WT neurons (Stobrawa et al., 2001). These experiments were performed on acute slices from *Clcn3-/-* animals at developmental stages at which degenerative changes are already occurring and at which expression levels of VGLUT (VGLUT1) were found to be decreased (Stobrawa et al., 2001). The differences between these and our results are likely due to a neurodegeneration-associated reduction in VGLUT1 expression.

ClC-3 was reported to function as voltage-dependent anion-proton exchangers by several groups (Li et al., 2002; Guzman et al., 2013). Since ClC-3 is expressed in SV (Stobrawa et al., 2001; Gronborg et al., 2010), lack of ClC-3-mediated chloride-proton exchanger might reduce vesicular counter ion movement and result in increased depolarization of SV by electrogenic V-type ATPases. ClC-3 exhibits a large probability of incomplete transport cycle resulting in prominent capacitive current transients at voltages in the range of synaptic vesicle potentials (Guzman et al., 2013). This property will result in larger vesicular capacitances and therefore in less depolarized vesicular membrane potentials of WT than of *Clcn3-/-* neurons. Chloride/proton exchange as well as capacitor function of ClC-3 will diminish the driving force for VGLUTs (Guzman et al., 2013) that are mainly driven by the vesicular membrane potential (Maycox et al., 1988). These effects are consistent with the observed increase of glutamate accumulation in *Clcn3-/-* SV.

The neurotransmitter content of SV is in dynamic equilibrium between transporter-mediated accumulation and leakage (Williams, 1997; Takamori, 2006). Genetic ablation of ClC-3 might enhance the osmotic gradient across the vesicular membrane via stimulating neurotransmitter accumulation and thus cause water influx and vesicular growth (Colliver et al., 2000; Pothos et al., 2000; Daniels et al., 2004). Increased glutamate accumulation and water influx into SV will reach a new equilibrium at higher values for both parameters and thus account for the observed difference in vesicle volume between WT and *Clcn3-/-* neurons.

We observed a twofold increase mEPSC frequency in *Clcn3-/-* that was not a consequence of an enhanced synaptic density (**Figures 5F,G**). Reduction in paired pulse ratio (**Figures 3E,F**) together with the unchanged size of RRP in *Clcn3-/-* neuronal cultures (**Figures 3C,D**) indicates that genetic ablation of ClC-3 proteins does not interfere with the establishment of the vesicle’s release-ready state. A possible explanation for the increased likelihood of synaptic vesicle fusion observed in *Clcn3-/-* synapses might be synaptic vesicle swelling due to increased glutamate accumulation. For many years, it has been known that osmotic swelling is a driving force for the fusion of vesicles with planar bilayers (Cohen et al., 1982; Akabas et al., 1984). It is tempting to speculate that enhanced glutamate accumulation might directly result in increased vesicle fusion in *Clcn3-/-*neurons via osmotic swelling. Alternatively, ClC-3 might regulate vesicle fusion via – yet to be defined - interacting protein partners in the presynaptic nerve terminal.

Our conclusion that ClC-3 restricts glutamate accumulation in SV could be further substantiated by rescuing experiments with *Clcn3-/-* neurons that express ClC-3 after viral infection. Unfortunately, the existence of multiple splice ClC-3 variants with probably distinct function and localization (Ogura et al., 2002; Gentzsch et al., 2003; Guzman et al., 2013) makes such experiments currently unfeasible. The identification of all ClC-3 splice variants together with their corresponding subcellular distribution will be an important step to further understand the precise role of this CLC isoform in synaptic transmission.

Our results demonstrate that genetic ablation of ClC-3 enhances the driving force for vesicular glutamate accumulation and thus assign a presynaptic role in regulating the vesicular glutamate concentration to ClC-3. However, there are also reports on postsynaptic localization of this protein. Wang et al. (2006) postulated that ClC-3 forms a postsynaptic CaMKII-activated chloride channel. ClC-3 functioning as anion channels was postulated to modify neuronal excitability by providing a regulated postsynaptic anion conductance that protects form excessive Ca^2^^+^ influx via NMDA receptors (Wang et al., 2006; Farmer et al., 2012). The authors speculated that absent ClC-3 increases NMDA signals even when presynaptic glutamate release is reduced (Stobrawa et al., 2001) and thus results in neurodegeneration in *Clcn3-/-* mice. There are different ClC-3 splice variants that may have multiple functional properties and localize to distinct neuronal compartments (Ogura et al., 2002; Gentzsch et al., 2003). Although our data strongly support presynaptic localization and anion-proton exchanger function of at least certain ClC-3 splice variants (Guzman et al., 2013), it is possible that other splice variants may function as anion channels in the postsynaptic compartments.

In conclusion, we here demonstrate that ClC-3 proteins modulate synaptic strength at glutamatergic synapses by regulating the amount of neurotransmitter stored in a single synaptic vesicle as well as its likelihood of fusion. These findings assign a presynaptic role in regulating glutamate release to ClC-3. Our results suggest that ClC-3 functions as safety measure to prevent glutamate excitotoxicity in WT animals and that excessive release of glutamate contributes to neurodegeneration in *Clcn3-/-* mice.

## AUTHOR CONTRIBUTIONS

Raul E. Guzman and Christoph Fahlke designed research; Raul E. Guzman, Alexi K. Alekov, Mikhail Filippov, Jan Hegermann performed research; Raul E. Guzman, Alexi K. Alekov, Jan Hegermann analyzed data; and Raul E. Guzman and Christoph Fahlke wrote the paper.

## Conflict of Interest Statement

The authors declare that the research was conducted in the absence of any commercial or financial relationships that could be construed as a potential conflict of interest.
